# 肺癌靶向药物肝脏毒性作用研究进展

**DOI:** 10.3779/j.issn.1009-3419.2014.09.08

**Published:** 2014-09-20

**Authors:** 雪琴 陈

**Affiliations:** 310006 杭州，南京医科大学附属杭州医院（杭州市第一人民医院集团）肿瘤内科 Department of Medical Oncology, Afliated Hangzhou Hospital (Hangzhou First People's Hospital), Nanjing Medical University, Hangzhou 310006, China

**Keywords:** 肺肿瘤, 靶向药物, 肝脏毒性, Lung neoplasms, Targeted drugs, Hepatotoxicity

## Abstract

针对驱动基因的靶向药物吉非替尼、厄洛替尼及克唑替尼等在晚期非小细胞肺癌治疗中有着不可替代的地位，然而此类药物给患者带来益处的同时也出现较高的肝脏毒性，现就其肝脏毒性及机制作一综述。

## Abstract (en)

Targeted drugs aimed at driver gene, such as Geftinib, Erlotinib and Crizotinib, have an irreplaceable position in the therapy of advanced non-small cell lung cancer. Tese drugs bring beneft to patients, however, higher hepatotoxicity is also presented. Now, drug induced hepatotoxicity and its mechanism are reviewed.

肺癌是全球发病率和死亡率最高的恶性肿瘤之一，约80%-85%为非小细胞肺癌(non-small cell lung cancer, NSCLC) ^[1]^，而且80%患者为局部晚期(Ⅲa期/Ⅲb期)或转移性(Ⅳ期)肺癌，预后极差，据报道5年生存率Ⅲa期和Ⅲb期/Ⅳ期分别为8%-14%和1%-5%^[2]^。晚期NSCLC的标准治疗方案——以铂类为基础的两药化疗疗效已到了瓶颈期，个体化治疗是当前研究的热点，某些已明确的或潜在的靶点即所谓的驱动基因是肿瘤治疗的依据。最近，美国肺癌突变协会的研究^[3]^提示NSCLC中最常见的驱动基因有*KRAS*突变25%、*EGFR*敏感突变17%、*ALK*基因突变8%、*EGFR*其他类型突变4%、*HER2*突变3%、*BRAF*突变2%等。而中国人群的驱动基因有所差异，*EGFR*敏感突变24.5%、*KRAS*突变2.88%、*ALK*基因突变3.37% ^[4]^。目前用于治疗晚期NSCLC的靶向药物主要包括吉非替尼(Gefitinib)、厄洛替尼(Erlotinib)、阿法替尼(Afatinib)及克唑替尼(Crizotinib)等，此类小分子化合物有疗效佳、耐受性好的优点，但是其肝脏毒性仍不容忽视，现就其肝脏毒性及机制综述如下。

## 1 肺癌靶向药物肝脏毒性概述

### 1.1 Geftinib和Erlotinib的肝脏毒性

Geftinib和Erlotinib是一种选择性表皮生长因子受体酪氨酸激酶抑制剂(epithelial growth factor receptor tyrosine kinase inhibition, EGFR-TKI)，主要通过抑制酪氨酸激酶阻断细胞内的信号传导通路，从而诱导肿瘤细胞凋亡。近几年的临床研究^[5-8]^显示Gefitinib和Erlotinib一线治疗*EGFR*突变的晚期NSCLC缓解率(response rate, RR)为62.1%-84.6%，无进展生存(progression-free survival, PFS)为8.4个月-13.1个月，明显高于化疗组(32.2%-47.3%和4.6个月-6.7个月)。TKI给这部分患者带来临床获益的同时，随着生存的延长，毒副反应也相应增加。多项研究^[9-11]^显示对于*EGFR*基因突变不明的NSCLC患者接受Gefitinib治疗后谷丙转氨酶(alanine aminotransferase, ALT)和谷草转氨酶(aspartate aminotransferase, AST)升高分别为21%-30%和14%-30%，且3级-4级升高为2%-5%，其中AST 3级-4级升高达11.33%；而获益更多的*EGFR*突变的NSCLC患者则不良反应更高，文献^[6]^报道ALT和AST最高均达72.6%，其中3级-4级分别为28.6%和16.7%。但胆红素升高及临床症状均未见报道。也有学者^[12]^报道使用Gefitinib治疗26例NSCLC患者后，39%患者出现肝功能损害，但未发现*EGFR*突变状况与肝功能损害发生频率及严重级别有关系。

Erlotinib对于西方人种和亚裔人种所致肝脏毒性似乎有所差异。西班牙EURTAC研究^[13]^提示*EGFR*基因突变的西方晚期NSCLC患者经Erlotinib治疗后所有级别的肝酶升高发生率为6%，3级发生率为2%；而OPTIMAL研究提示接受Erlotinib治疗EGFR基因突变的中国NSCLC患者ALT升高率为37%，其中3级-4级为4%^[8]^。此外，有日本学者^[14]^回顾性分析了Gefitinib和Erlotinib治疗NSCLC不良反应的差异，发现前者肝脏损害作用发生率高于后者，但未见统计学差异。以上研究均是临床研究的药物不良事件分析或小样本研究，还有待于更大规模的荟萃分析。

### 1.2 Crizotinib的肝脏毒性

Crizotinib是一种口服的、针对间变性淋巴瘤激酶(anaplastic lymphoma kinase, *ALK*)基因重组、*c-MET*基因扩增和*ROS*基因重组靶点的小分子三磷酸腺苷竞争性的多靶点酪氨酸激酶受体抑制剂。目前美国食品药品监督管理局(Food and Drug Administration, FDA)和中国食品药品监督管理局已批准Crizotinib用于ALK基因阳性的局部晚期或转移性NSCLC患者的治疗。Ⅰ期、Ⅱ期临床研究PROFILE 1001^[15]^和PROFILE 1005^[16]^提示，对于ALK阳性的进展期NSCLC患者，Crizotinib的客观有效率分别为61%和60%，PFS分别为9.7个月和8.1个月。PROFILE1007研究^[17]^提示Crizotinib二线治疗ALK阳性的NSCLC患者，PFS(7.7个月)和RR(65%)明显优于化疗组的3.0个月和20%。但是其肝脏毒性也值得重视，PROFILE 1001和PROFILE 1005研究^[18]^发现Crizotinib导致ALT和AST升高分别为14%和10%，其中3级-4级毒性分别为5%和2%；Ⅲ期临床研究PROFILE1007^[17]^提示Crizotinib的转氨酶升高发生率为38%，3级-4级发生率为16%，主要表现为无症状的肝酶升高，合并胆红素升高罕见。

### 1.3 Afatinib的肝脏毒性

Afatinib是一种口服EGFR-TKI的不可逆抑制剂。Lux-lung Ⅲ期研究^[19]^提示Afatinib一线治疗*EGFR*突变的进展期NSCLC患者PFS和RR明显优于培美曲塞联合顺铂组；Ⅱ期临床研究^[20]^显示，具有*EGFR*突变的NSCLC患者，对第一代TKI耐药时，Afatinib仍对其有抗瘤活性的潜力。但这几项临床研究^[19-21]^中均未见到转氨酶明显升高的报告，提示所有级别的肝酶升高发生率 < 10%，可见Afatinib的肝脏损害作用并不明显。

### 1.4 达沙替尼的肝脏毒性

达沙替尼(Dasatinib)是一种新型的经FDA批准治疗慢性粒细胞性白血病的小分子多靶点酪氨酸激酶抑制剂，对多种受体的酪氨酸激酶具有抑制作用。Hochhaus等^[22]^在对186例慢性粒细胞白血病患者服用Dasatinib为期8个月的跟踪调查中发现96例(52%)出现ALT升高，其中3例(2%)升高程度达到3或4级，111例(60%)出现AST升高，其中4例(2%)升高程度达到3级或4级。近来，Johnson报道^[23]^Dasatinib治疗进展期NSCLC Ⅱ期临床研究，其中1例患者有明显疗效、另外4例患者长时间维持疾病稳定，这提示有潜在敏感的亚组人群，尤其在目前EGFR-TKI相对不敏感的鳞癌患者中不失为一种希望。故其肝脏毒性也值得关注。

此外，Teo等^[24]^进行了一项关于癌症患者TKI肝脏毒性风险的*meta*分析，分析了20项随机、双盲、安慰剂对照的Ⅱ期或Ⅲ期临床研究，涵盖2000年1月后经FDA批准的Gefitinib、Erlotinib、Crizotinib、Dasatinib、索拉非尼、舒尼替尼、伊马替尼等14种TKI，结果发现应用TKI后3级/4级肝毒性明显高于对照组(HR=4.35, 95%CI: 2.96-6.39)；所有级别发生肝毒性的HR=2.42(95%CI: 1.52-3.85)，其中ALT为5.22(95%CI: 2.88-9.46)，AST为6.15(95%CI: 3.09-12.25)，总胆红素为1.76(95%CI: 0.59-5.24)。这项研究提示临床工作者应该警惕这种风险，并密切监测接受TKI治疗患者的肝功能。

## 2 TKI肝脏毒性的机制

药物性肝损伤发生的机制尚未完全阐明，目前认为其发病机制可能主要通过三个途径：①药物及其代谢产物直接引起细胞应激(内源性途径)；②直接损伤线粒体β氧化和呼吸链功能；③激活特异免疫反应(外源性途径)。从而导致线粒体通透性改变，甚至线粒体破裂，最终引起肝细胞的死亡或凋亡^[25, 26]^。

肝细胞氧化应激是指活性氧(reactive oxygen species, ROS)产生过多或/和内源性抗氧化能力降低，氧化系统和抗氧化系统平衡紊乱，而致肝细胞损伤的病理过程。有丝分裂素激活蛋白激酶(mitogen-activated protein kinase, MAPK)是一类丝氨酸/苏氨酸激酶，MAPK信号通路参与细胞生长、发育、分裂等多种生理过程，并在细胞应激、恶性转化等病理过程也起重要作用。MAPK包括：细胞外信号调节的蛋白激酶、C-Jun N端激酶(JNK) /应激激活的蛋白激酶、p38 MAPK。JNK通路和P38通路能被促炎细胞因子和炎症反应介质等激活。文献^[26]^报道Dasatinib和Gefitinib作用后可引起细胞应激，消耗肝细胞内的谷胱甘肽，升高ROS水平，激活MAPK信号通路，激活核细胞因子E2相关因子2(nuclear factor erythroid 2-related factor 2, Nrf2)，可激活JNK和P38激酶，从而引起肝细胞凋亡；此外作者还发现Dasatinib与Gefitinib能诱导肝细胞产生保护性的自噬。

## 3 TKI肝脏毒性与基因多态性

TKI的肝脏毒性作用除了与细胞应激、免疫反应相关外，近年来也认为与药物代谢酶细胞色素P450 (cytochromep 450, CYP450)的基因多态性有关。CYP2D6是CYP酶家族重要成员之一，编码CYP2D6的基因位于第22号染色体，有9个外显子，8个内含子，共编码497种氨基酸。目前发现CYP2D6约有80个突变位点，基因突变可导致酶活性和数量的不同，引起药物代谢个体的差异，最后引起药物疗效或毒副作用的差异^[27]^。按基因突变体产物的功能可分为功能基因、功能缺陷基因和无功能基因。携带等位基因CYP2D6*5因大段基因缺失，导致整个CYP2D6功能的缺失，使酶没有活性，称无功能等位基因。含有CYP2D6*10的纯合子或杂合子，编码的蛋白质结构不稳定，使酶活性明显下降，称功能缺陷等位基因。文献^[28]^报道17例曾经服用Geftinib后发生肝脏毒性的肺癌患者，携带等位基因CYP2D6*5或*10者可以成功地转换到Erlotinib治疗(*P*=0.024)，但没有含这两个等位基因的患者则不能，提示那些曾经因Geftinib所致肝脏毒性且CYP2D6活性较弱的患者可以安全地使用Erlotinib。应用CYP3A4抑制剂后，7例携带CYP2D6*5或*10的患者再次使用Gefitinib仍引起肝脏毒性，而3例有正常等位基因的患者则不会发生肝脏毒性。提示CYP3A4被抑制后，CYP2D6酶功能的缺陷可能是Gefitnib引起肝脏毒性的主要原因。

## 4 TKI肝脏损伤后治疗策略

迄今为止，关于TKI导致肝脏损害后治疗的研究报道较少，只有少数的回顾性报道或个例报道。这些报道建议减少剂量，更改治疗策略如与化疗序贯，或转换为另一种TKI。文献^[29]^报道1例日本61岁女性肺腺癌患者口服Gefitinib 250 mg每日一次8周后疗效为完全缓解，但出现肝功能2级-3级损害(排外肝转移及其他肝病)，停止给药后导致疾病进展，再次使用后继续出现肝功能3级损害，故改为吉非替尼250 mg每5天一次，疗效完全缓解，肝功能1级损害。由此可见，TKI治疗后出现肝功能损害，可改变治疗剂量，仍然可以使患者获益。我们曾报道1例女性*EGFR*突变的肺腺癌患者服用Gefitinib治疗14个月后出现肝功能4级损害，停止用药给予护肝治疗2个月后肝功能好转至1级损害，但肺部病灶进展，给予化疗控制病情，待肝功能恢复至正常后，再次使用Geftinib靶向治疗并护肝治疗，随访半年病情稳定，肝酶正常^[30]^。此外，也有文献^[31]^报道2例NSCLC患者经吉非替尼治疗后导致2级/3级肝功能损害，均成功更换到另一种TKI如Erlotinib，其中1例经Erlotinib治疗后病灶达完全缓解，而且长时间维持。

## 5 小结及展望

肺癌患者应用TKI靶向药物肝脏毒性发生率较高，期间应定期复查，可以早期发现肝功能异常及时进行护肝治疗，避免过早盲目停药。由于个体遗传差异，药效及毒性反应也会迥然不同，单核苷酸基因多态性可能是预测指标之一，值得深入研究。此外，TKI所致肝脏毒性机制及护肝策略也有待于进一步研究。
